# Association Between Sensorineural Hearing Loss and Neurocognitive Performance in Survivors of Childhood Cancer: A Systematic Review and Meta‐Analysis

**DOI:** 10.1002/cam4.71394

**Published:** 2025-11-20

**Authors:** Jennifer E. Schlak, Tripti Shukla, Anne Eaton, Lindsay Blake, Alicia Kunin‐Batson, Ellen van der Plas

**Affiliations:** ^1^ Clinical Behavioral Neuroscience University of Minnesota Minneapolis Minnesota USA; ^2^ University of Arkansas for Medical Sciences Little Rock Arkansas USA; ^3^ Division of Biostatistics & Health Data Science University of Minnesota Minneapolis Minnesota USA; ^4^ Division of Research and Evidence‐Based Medicine University of Arkansas for Medical Sciences Little Rock Arkansas USA; ^5^ Department of Pediatrics University of Arkansas for Medical Sciences Little Rock Arkansas USA; ^6^ Division of Hematology/Oncology Arkansas Children's Hospital Little Rock Arkansas USA

**Keywords:** childhood cancer survivors, neurocognitive performance, neuropsychological tests, platinum‐based chemotherapy, radiation, sensorineural hearing loss

## Abstract

**Background:**

Various studies have shown that sensorineural hearing loss (SNHL) is associated with neurocognitive impairment among childhood cancer survivors, though prior studies are limited by small sample sizes and inconsistent methods.

**Objective:**

This systematic review and meta‐analysis aimed to quantify neurocognitive differences between survivors with severe SNHL (s‐SNHL) and those without.

**Methods:**

Studies were included if they evaluated childhood cancer survivors (diagnosed ≤ 21 years), were ≥ 2 years posttreatment, received cranial radiation and/or platinum‐based chemotherapy, used validated neurocognitive tests, and included survivors with and without SNHL. Methods and reporting adhered to the Preferred Reporting Items for Systematic Reviews and Meta‐Analyses (PRISMA) guidelines. Databases searched included PubMed, Web of Science, Cochrane, Scopus, PsycINFO, and Embase. Study quality was assessed using an adapted version of the Newcastle–Ottawa Scale. We collected study details, including authors, year, neurocognitive tests used, sample sizes, and group scores (means and standard deviations [SD]). Pooled mean differences (MD) and 95% confidence intervals (95% CI) were calculated using standard scores (mean = 100, SD = 15) and random‐effects models.

**Results:**

Of 1012 records identified, nine studies were included in the analyses, with 503 childhood cancer survivors with s‐SNHL and 1553 survivors without s‐SNHL. Compared to survivors without s‐SNHL, survivors with s‐SNHL scored lower on measures of overall intellectual functioning (MD = −8.1, 95% CI = −10.0, −6.2), verbal reasoning (MD = −9.7, 95% CI = −11.4, −7.9), perceptual reasoning (MD = −6.6, 95% CI = −11.1, −2.1), working memory (MD = −5.3, 95% CI = −7.1, −3.4), processing speed (MD = −6.3, 95% CI = −9.9, −2.6), short‐term visual memory (MD = −6.0, 95% CI = −8.2, −3.7), and reading (MD = −6.2, 95% CI = −7.9, −4.5).

**Conclusions:**

Severe SNHL is associated with significant neurocognitive deficits in childhood cancer survivors. Routine hearing screening and timely neurocognitive assessments are recommended to identify and address these impairments.

**Trial Registration:**

PROSPERO: CRD42023440952

## Graphical abstract

This systematic review and meta‐analysis found that childhood cancer survivors with severe sensorineural hearing loss (s‐SNHL) had significantly lower scores in various neurocognitive domains, including IQ, memory, and processing speed, compared to survivors without s‐SNHL. The findings highlight the importance of neuropsychological assessments for those with s‐SNHL to identify and address potential deficits in childhood cancer survivors.

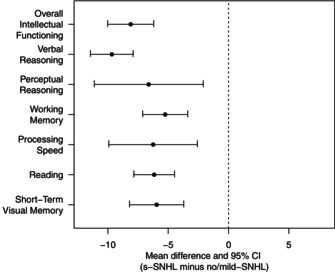

## 1 Introduction

As the number of childhood cancer survivors continues to grow, it is increasingly important to address the long‐term side effects of lifesaving cancer treatments, such as chemotherapy and radiation. A major long‐term side effect of cancer treatment is ototoxicity, which is damage to the ear that causes permanent sensorineuronal hearing loss (SNHL). Cranial radiation and/or platinum‐based chemotherapy agents, such as cisplatin and carboplatin have been identified as risk factors of SNHL [1, 2]. Radiation therapy is known to damage cochlear hair cells and the stria vascularis through direct structural injury, impairing ion balance and sound signal conversion [3], while platinum chemotherapy causes oxidative stress and inflammation by generating reactive oxygen species that lead to hair cell death [1, 4]. The estimated incidence of SNHL among survivors of central nervous system malignancies ranges between 10%–45%. This variability is primarily driven by differences in sample size and ototoxicity grading across studies [2, 5, 6, 7, 8].

SNHL is increasingly recognized as a notable risk factor for neurocognitive impairment among childhood cancer survivors. For example, survivors with SNHL showed lower overall cognitive abilities, such as full‐scale IQ, compared to those without hearing loss [3, 9]. Others have demonstrated deficits in verbal reasoning, verbal fluency, executive dysfunction, and memory among survivors with SNHL [3, 10, 11, 12]. SNHL further hinders academic performance and language development, with survivors struggling in reading comprehension, word reading, and math skills [3, 13]. Both SNHL and neurocognitive impairment have been identified as risk factors for poor quality of life for childhood cancer survivors [14, 15, 16]. These findings underscore the necessity of continued research to determine evidence‐based best practices for treatment and intervention.

While the literature on associations between SNHL and neurocognitive impairment in childhood cancer survivors is growing, studies are typically relatively small, and in particular, subgroups with severe SNHL tend to be small [13, 17], limiting power. The ability to generalize results is further limited by the use of different cognitive measures across studies. The variability in studies is compounded by the use of different hearing loss classifications, the inclusion of various treatment approaches, and the examination of distinct patient populations. By synthesizing data from multiple sources, a meta‐analysis can provide a more comprehensive and precise evaluation of the true impact of SNHL on neurocognitive outcomes across diverse cancer types and treatment protocols. This approach allows for a better understanding of the effects of treatment‐induced SNHL, offering a more consistent foundation for future research and clinical interventions. The aim of the present study was to summarize the literature and quantify the impact of severe SNHL (s‐SNHL) on neurocognitive function among childhood cancer survivors by comparing neurocognitive task performance between childhood cancer survivors with and without s‐SNHL.

## 2 Methods

### 2.1 Protocol and Search Strategy

**FIGURE 1:**
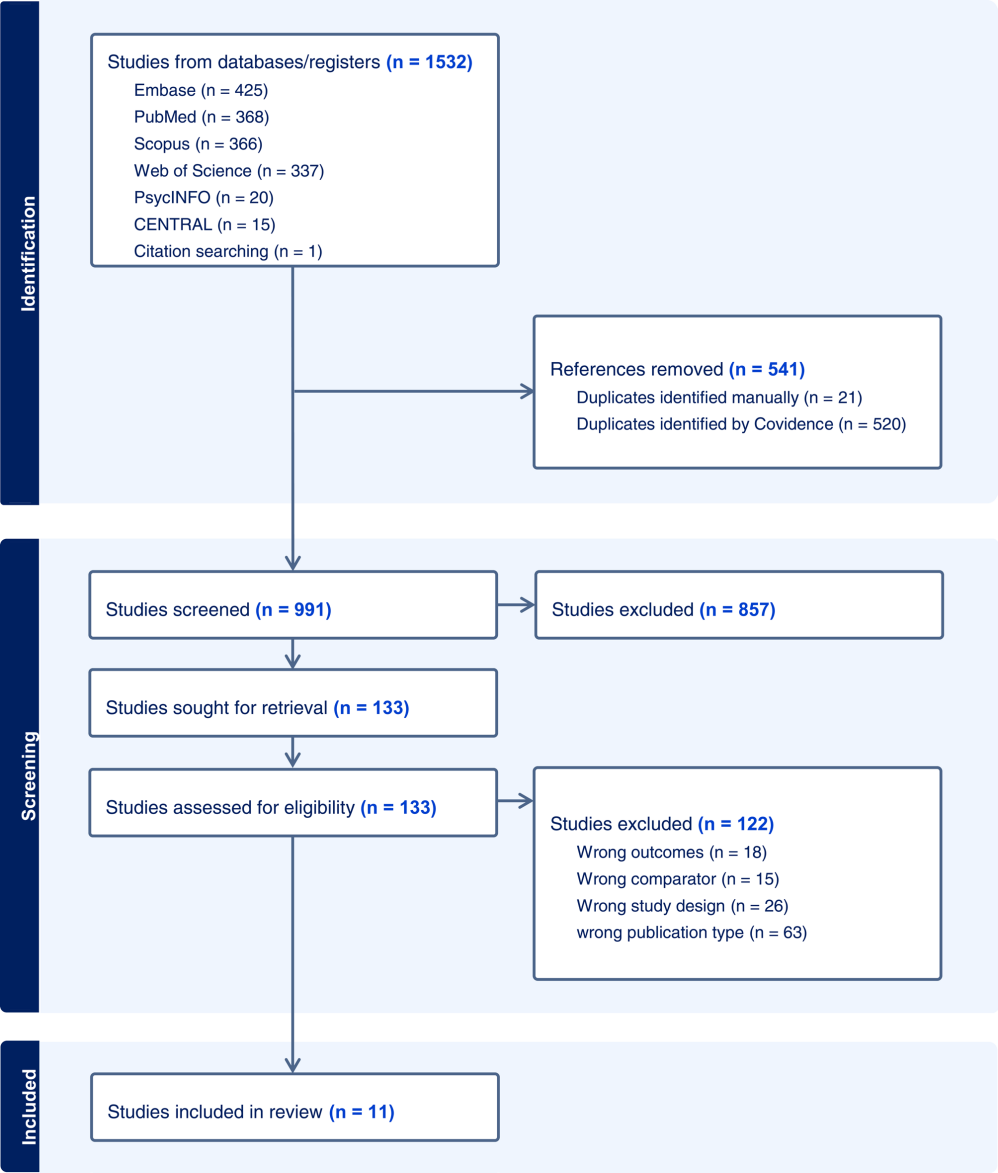
PRISMA flow chart of study selection.

The review and meta‐analysis are registered in PROSPERO. The study was conducted in accordance with the Preferred Reporting Items for Systematic Reviews and Meta‐Analyses (PRISMA). The literature was searched through the citation databases: PubMed for MEDLINE, Web of Science, Cochrane Database of Systematic Reviews, Scopus, PsycINFO and Embase on June 29th, 2023. A gray literature search for clinical trials was conducted in the Cochrane Central Register of Controlled Trials. Databases were searched with both keywords and controlled vocabulary (MeSH, etc.) when available, around the topics of ototoxicity, antineoplastic agents, childhood cancers or cancer survivors, and neurocognitive development (Table S1). Searches yielded 869 citations after duplicates were removed. Abstracts were reviewed by two authors for inclusion. Full‐text articles were retrieved and reviewed for inclusion by two authors for 122 articles, resulting in 10 articles for data extraction. Subsequent searches were conducted on August 6th, 2024 and July 23, 2025, to ensure recently published articles were found. An additional 143 citations were reviewed using the same methods described above and one article was added to the data extraction. Figure 1 summarizes the selection process that resulted in 11 articles from nine studies being included in the final analysis.

### 2.2 Eligibility Criteria

The following criteria were considered for studies that evaluated neurocognitive function in childhood cancer survivors: (1) childhood cancer survivors who were ≤ 21 years old at diagnosis; (2) exposed to cranial radiation and/or platinum‐based chemotherapy; (3) survivors who were ≥ 2 years posttreatment; (4) the study used validated, standardized performance‐based neurocognitive measures; (5) the study design included survivors with and without SNHL; and (6) a sample size of ≥ 20 participants. Study designs to be considered for review included original research articles, observational studies, prospective and retrospective cohort studies, case‐control studies, cross‐sectional studies, longitudinal studies, randomized trials, systematic reviews, and meta‐analyses. The latter were included to identify potentially eligible studies, but meta‐analyses themselves were not included in the computation. Narrative review articles, conference papers, abstracts, posters, dissertations, editorials, letters to the editor, and case studies or case series were excluded. Studies were reviewed by two investigators and any discrepancies between authors were resolved through consensus discussion.

### 2.3 Data Extraction

Data on authors and publication year, neurocognitive instrument(s), number of survivors, and means and standard deviations for each neurocognitive measure across groups were extracted. We approached study authors to obtain means and standard deviations when these statistics were not available in the published materials [3, 12, 18].

### 2.4 Independent Variable

Presence of s‐SNHL (yes vs. no) served as the independent variable across analyses. To determine the presence of s‐SNHL, studies typically extracted ototoxicity profiles from audiograms and assigned a grade based on validated ototoxicity grading scales. s‐SNHL was defined as either Chang grade ≥ 2b [3, 9, 18, 19, 20, 21], Boston Ototoxicity Scale grade 3 or 4 [17], or Brock Grade 3 or 4 [10]. Although different grading systems were used across studies, the decibel (dB) and hertz (Hz) parameters used to define s‐SNHL were within similar ranges (Table S2). One study defined severe hearing loss using the Common Terminology Criteria for Adverse Events grades 2–4 [12]. A modified version of the Newcastle‐Ottawa Quality Assessment scale for cross‐sectional studies [22] was used to assess the quality of the studies included in the meta‐analysis (Table S3).

### 2.5 Neurocognitive Outcomes

We reviewed the instruments used in each study and classified each instrument according to the neurocognitive domain being measured. Neurocognitive domains included in the current analysis were those that were measured in four or more studies. We did not report on neurocognitive domains measured in three or fewer studies due to inadequate sampling. When a study presented results from multiple instruments within a given domain, we selected the instrument that achieved consensus for being most aligned with those used in other studies. As a result, each study contributed at most one score for each given domain. Table 1 provides an overview of the instruments used to measure each domain. For easier interpretation, scores that were reported as *Z*‐scores, *T*‐scores or scaled scores were converted to standard scores (mean = 100, standard deviation = 15, higher score indicates better performance). Seven outcomes, described briefly below, were included in the analyses.

**TABLE 1:** Cognitive domains and measures across included studies.

	Study								
	Bass	Fay‐McClymont	Heitzer	L'Hotta	Moxon‐Embre	Notteghem	Orgel	Tonning Olsson	Conklin
Overall intellectual function									
Outcome	FSIQ	FSIQ	FSIQ	Total composite	FSIQ	N/A	FSIQ	N/A	FSIQ
Measure(s)	WASI; WASI‐II	DAS‐II; Leiter‐R; RIAS; UNIT; WPPSI‐III, IV; WISC‐IV; WJ‐III Cog	WAIS‐IV; WISC‐IV, V; WPPSI‐III, IV	NIH TB	WASI; WAIS‐IV; WISC‐III, IV, V; WPPSI‐III, IV; WJ‐III Cog	N/A	WASI WAIS‐III, IV; WISC‐III, IV; WPPSI‐R, III;	N/A	SB‐5
Verbal reasoning									
Outcome	Vocabulary	VIQ	VCI	Language‐ vocabulary	Verbal comprehension	VIQ	VCI	Verbal reasoning	N/A
Measure(s)	WASI; WASI‐II	DAS‐II; RIAS; UNIT; WPPSI‐III, IV; WISC‐IV; WJ‐III Cog	WAIS‐IV; WISC‐IV, V; WPPSI‐III, IV	NIH TB	WASI; WAIS‐IV; WISC‐III, IV, V; WPPSI‐III, IV; WJ‐III Cog	WAIS‐III; WISC‐III; WPPSI‐R	WASI WAIS‐III, IV; WISC‐III, IV; WPPSI‐R, III;	WASI	N/A
Perceptual reasoning									
Outcome	N/A	PIQ	PRI	N/A	Perceptual reasoning	PIQ	PRI	Matrix reasoning	N/A
Measure(s)	N/A	DAS‐II; Leiter‐R; RIAS; UNIT; WPPSI‐III, IV; WISC‐IV; WJ‐III Cog	WAIS‐IV; WISC‐IV, V; WPPSI‐III, IV	N/A	WASI; WAIS‐IV; WISC‐III, IV, V; WPPSI‐III, IV; WJ‐III Cog	WAIS‐III; WISC‐III; WPPSI‐R	WASI WAIS‐III, IV; WISC‐III, IV; WPPSI‐R, III;	WASI	N/A
Working memory									
Outcome	Digit span backward	Working memory	WMI	Working memory	Working memory	N/A	WMI	Digit span backward	N/A
Measure(s)	Wechsler Intelligence Scales	DAS‐II; WPPSI‐III, IV; WISC‐IV; WJ‐III Cog	WAIS‐IV; WISC‐IV, V; WPPSI‐III, IV	NIH TB	WASI; WAIS‐IV; WISC‐III, IV, V; WPPSI‐III, IV; WJ‐III Cog	N/A	WASI WAIS‐III, IV; WISC‐III, IV; WPPSI‐R, III;	WASI	N/A
Processing speed									
Outcome	Digit symbol‐coding	Pro‐cessing speed	PSI	Processing speed	Processing speed	N/A	PSI	Symbol search	N/A
Measure(s)	Wechsler Intelligence Scales	DAS‐II; WPPSI‐III, IV; WISC‐IV; WJ‐III Cog	WAIS‐IV; WISC‐IV, V; WPPSI‐III, IV	NIH TB	WASI; WAIS‐IV; WISC‐III, IV, V; WPPSI‐III, IV; WJ‐III Cog	N/A	WASI WAIS‐III, IV; WISC‐III, IV; WPPSI‐R, III;	Wechsler Intelligence Scales	N/A
Reading									
Outcome	Letter‐word identification	N/A	N/A	Language‐oral reading	N/A	N/A	Single word reading	Letter word identification	N/A
Measure(s)	WJ III Ach	N/A	N/A	NIH TB	N/A	N/A	WIAT‐II, III; WRAT‐III, IV; WJ‐III Ach	WJ Ach	N/A
Short‐term visual memory									
Outcome	Visual selective reminding	N/A	N/A	Episodic memory	N/A	N/A	Visual memory—immediate	Visual selective reminding	N/A
Measure(s)	TOMAL‐II	N/A	N/A	NIH TB	N/A	N/A	CMS; WMS‐R, III, IV; WRAML I, II;	TOMAL‐II	N/A

Abbreviations: CMS, Children's Memory Scale; DAS, Differential Abilities Scale; FSIQ, Full Scale Intellectual Quotient; Leiter, Leiter International Performance Scale; NIH TB, National Institute of Health Toolbox; PIQ, Performance Intelligence Quotient; PRI, Perceptual Reasoning Index; PSI, Processing Speed Index; RIAS, Reynolds Intellectual Assessment Scale; SB‐5, Stanford‐Binet Intelligence Scales, Fifth Edition; TOMAL, Test of Memory and Learning; UNIT, Universal Nonverbal Intelligence Test; VCI, Verbal Comprehension Index; VIQ, Verbal intelligence Quotient; WAIS, Wechsler Adult Intelligence Scale; WASI, Wechsler Abbreviated Scale of Intelligence; WIAT, Wechsler Individual Achievement Test; WISC, Wechsler Intelligence Scale for Children; WJ Ach, Woodcock‐Johnson, Tests of Achievement; WJ Cog, Woodcock‐Johnson, Tests of Cognitive Abilities; WMI, Working Memory Index; Wechsler Intelligence Scales, versions not specified; WMS, Wechsler Memory Scale; WPPSI, Wechsler Preschool and Primary Scale of Intelligence; WRAML, Wide Range Assessment of Memory and Learning; WRAT, Wide Range Achievement Test.

The first outcome, overall intellectual functioning, reflects an individual's general intellectual ability and represents a combination of various cognitive domains, such as problem‐solving, working memory, and processing speed abilities. The second outcome, verbal reasoning pertains to the ability to comprehend, analyze and solve problems through language‐based tasks. Next, we analyzed perceptual reasoning, which refers to the ability to understand and solve problems using visual and spatial information. Working memory, the fourth outcome, represents the ability to mentally hold and manipulate information. The fifth outcome, processing speed, measures the efficiency with which an individual processes simple information. Sixth, we assessed reading, which involves decoding and reading words aloud. Finally, short‐term visual memory, refers to the ability to remember visual information for a short period of time.

### 2.6 Statistical Approach

We used a random effects model and reported mean differences in standard score between the participants with s‐SNHL versus no severe SNHL, with 95% confidence intervals [23]. The model assumes two sources of variability in the observed effect sizes: between‐study variability (i.e., differences in the true effect size between studies due to differences in the population or other parts of the study design) and within‐study variability (variability between individuals in a study). True study‐specific effect sizes and errors (the differences between the observed and true study‐specific effect sizes) are assumed to be normally distributed. We examined funnel plots (the standard error of each study plotted by its mean difference) for evidence of publication bias. *I*‐squared statistics were used to assess heterogeneity across studies. For domains with *p* < 0.2 for the test of heterogeneity based on the *Q* statistic, sensitivity analyses were performed by removing one study at a time. The threshold of 0.2 was selected to increase the chance that a domain with true inter‐study heterogeneity would be explored further, at the cost of an increased risk (20%) of flagging a domain with no true inter‐study heterogeneity. All statistical analysis was performed using R 4.3.1 and the meta package was used [24]. *p*‐values < 0.05 were considered statistically significant.

## 3 Results

The final sample included 2056 childhood cancer survivors (910 females [45%]), where 503 survivors had s‐SNHL and 1553 did not. Mean age at diagnosis was 6.6 years old (SD = 4.9 years), and age at follow‐up was on average 17.7 years old (SD = 12.3 years). Mean age at diagnosis and follow‐up was calculated from all studies except Bass et al. and Notteghem et al., which reported medians and ranges instead. Table 2 shows how the quality for each of the nine studies was rated and Table 3 provides a summary of sample characteristics.

**TABLE 2:** Quality assessment of studies included in the meta‐analysis.

Newcastle Ottawa Scale	Selection			Comparability	Outcome		Total
	1	2	3	1	1	2	
Study ID	Representation	Sample size	Non‐included subjects	Groups are comparable	Clearly described, appropriate stats	Measures of associations presented with variability	
Bass	*	*	—	*	*	*	5/7
Fay‐Mclyont	—	—	*	*	*	*	4/7
L'hotta	*	—	—	*	*	*	4/7
Heitzer	*	*	—	**	*	*	6/7
Moxon‐Emre	—	—	—	**	*	*	4/7
Notteghem	*	—	—	*	*	*	4/7
Orgel	—	*	—	**	*	*	5/7
Tonning	*	—	—	*	*	*	4/7
Conklin	*	—	*	*	*	*	6/7

*Note:* See Table S3 for an overview of the point adjudication. The * represents points adjudicated for that item, where one “*” represents one point and two “**” represents 2 points.

**TABLE 3:** Key characteristics of the studies included in the meta‐analysis.

Study	Sample		SNHL status		Key cancer/treatment characteristics of the sample	
	*N* (%)/Mean/SD		*N* (%)		*N* (%); dose Mean/SD	
Bass	Total	1520	Severe	353 (23%)	CNS tumor	336 (22%)
	Female	695 (46%)	None/Mild	1167 (76%)	Non‐CNS tumor	1184 (78%)
	Time since dx, y	20^a^ (6–54)^b^			Carboplatin	188 (12%)
	Age FUP, y	29^a^ (7–65)^b^			Cisplatin	279 (18%)
	Tx Era	2007–2017			Cranial RT	379 (25%)
					Cranial RT, Gy	50^a^ (24–55)^b^
					Cochlear RT	473 (31%)
Fay‐McClymont	Total	24	Severe	7 (29%)	Nodular desmoplastic	16 (67%)
	Female	9 (38%)	None/Mild	17 (70%)	Metastatic at dx	7 (29%)
	Age dx, y	3 (1)			CS RT	4 (17%)
	Age FUP, y	6 (2)			Focal RT^d^	5 (21%)
	Tx Era	1998–2011				
L'Hotta	Total	52	Severe	6 (12%)	Neuroblastoma	16 (32%)
	Female	25 (48%)	None/Mild	46 (88%)	Germ cell tumor	13 (24%)
	Age dx, y	4 (4)			Retinoblastoma	10 (19%)
	Age FUP, y	12 (3)			Other	13 (25%)
	Tx Era	2005–2021			Cisplatin^e^	24 (46%)
					Cisplatin, mg/m^2^	469 (170)
					Carboplatin^e^	31 (60%)
					Carboplatin, mg/m^2^	2621 (1670)
Heitzer	Total	36	Severe	17 (47%)	Medulloblastoma	34 (94%)
	Female	8 (22%)	None/Mild	19 (53%)	Other	2 (6%)
	Age dx, y	7 (4)			Cisplatin	36 (100%)
	Age FUP, y	14 (3)			Cisplatin, mg/m^2^	305 (78)
	Tx Era	1996–2015			Cranial RT	36 (100%)
					Cranial RT, Gy	26 (1)
					Focal RT^d^	36 (100%)
					Focal RT, Gy	54 (4)
Moxon‐Emre	Total	94	Severe	48 (51%)	Medulloblastoma	77 (82%)
	Female	35 (37%)	None/Mild	46 (49%)	ATRT	9 (10%)
	Age dx, y	7 (4)			Pineoblastoma	7 (7%)
	Age FUP, y	11 (4)			PNET	1 (1%)
	Tx Era	1996–2016			CS RT	44 (47%)
					Reduced‐dose CS RT	34 (36%)
					Cisplatin, mg/m^2^	308 (109)
					Carboplatin, mg/m^2^	439 (955)
Notteghem	Total	76	Severe	8 (11%)	Neuroblastoma	46 (61%)
	Female	39 (51%)	None/Mild	68 (98%)	Ewing's Sarcoma	10 (13%)
	Age dx, y	4^a^ (0–20)^c^			Other	20 (26%)
	Age FUP, y	16^a^ (7–32)^c^				
	TX Era	1981–1995				
Orgel	Total	58	Severe	32 (55%)	Medulloblastoma	39 (67%)
	Female	22 (38%)	None/Mild	26 (45%)	Other CNS Tumor	19 (33%)
	Age dx, y	7 (1)			HSCT	20 (35%)
	Age FUP, y	11 (1)			Cisplatin	58 (100%)
	TX Era	~1988–~2015^f^			Cisplatinum, mg/m^2^	333 (16)
					Carboplatinum	19 (33%)
					Cranial RT	46 (79%)
					Cranial RT, Gy	29.5 (2)
Tonning	Total	150	Severe	22 (15%)	Rhabdomyosarcoma	91 (61%)
	Female	61 (41%)	None/Mild	128 (85%)	Non‐rhabdo STS	59 (39%)
	Age dx, y	9 (6)			Cranial RT	28 (19%)
	Age FUP, y	33 (9)			Cranial RT, Gy	4400 (1377)
	TX Era	2007–2019				
Conklin	Total	46	Severe	10 (22%)	Medulloblastoma	9 (19%)
	Female	16 (35%)	None/Mild	36 (78%)	ATRT	6 (13%)
	Age dx, y	1.5 (0.9)			PNET	5 (11%)
	Age FUP, y	5.2 (0.2)			Glioma, all types	3 (7%)
	Tx Era	2007–2017			Ependymoma, all types	12 (26%)
					Other	11 (24%)
					Chemo only	19 (41%)
					Focal Photon RT	10 (22%)
					Focal Proton RT	17 (37%)

Abbreviations: ATRT, atypical teratoid rhabdoid tumor; CNS, central nervous system; CS, craniospinal; Dx, diagnosis; FUP, follow‐up; HSCT, hematopoietic stem cell transplant; NOS, Newcastle‐Ottawa Quality Assessment Scale; PNET, primitive neuroectodermal tumor; RT, radiotherapy; STS, soft tissue sarcoma; TX, treatment; y, years.

^a^ Median.

^b^ IQR.

^c^ Min, max.

^d^ Radiation directed to the tumor bed.

^e^ Four patients received both carboplatin and cisplatin.

^f^ Approximated dates based on the reported treatment protocols.

**FIGURE 2:**
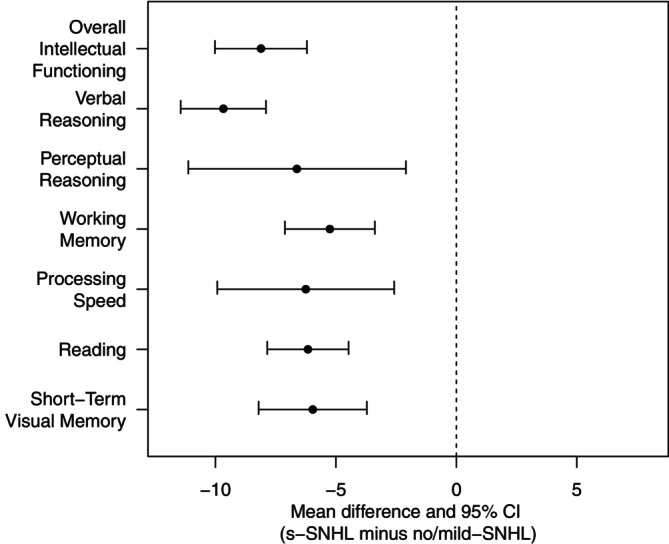
Forest plots for comparison of neurocognitive outcomes across survivors with and without SNHL. Mean differences and 95% confidence limits are shown across neurocognitive domains (*y*‐axis) for which ≥ 4 studies were available. The *x*‐axis shows the difference in standard scores with negative differences favoring the group without severe hearing loss and positive differences favoring the group with severe hearing loss. The vertical, dotted line marks 0, that is, no difference between the groups.

A summary of the findings of the meta‐analysis is provided in Table 4 and Figure 2. Forest and funnel plots for each of the neurocognitive domains are shown in the Data S1.

**TABLE 4:** Number of studies and pooled sample characteristics.

Outcome	Studies	*N* S‐SNHL	*N* No s‐SNHL	MD	95% CI	*p*	*I* ^2^
Overall intellectual functioning	7	457	1307	−8.11	−10.02, −6.21	< 0.001	4.3
Verbal reasoning	8	480	1477	−9.67	−11.44, −7.91	< 0.001	0
Perceptual reasoning	6	121	281	−6.62	−11.13, −2.10	< 0.01	38.5
Working memory	7	297	1084	−5.25	−7.12, −3.38	< 0.001	0
Processing speed	7	467	1393	−6.25	−9.92, −2.58	< 0.001	45.3
Reading	4	368	1279	−6.16	−7.85, −4.47	< 0.001	0
Short‐term visual memory	4	350	1268	−5.96	−8.21, −3.72	< 0.001	0

Abbreviations: CI, confidence interval; MD, mean difference; s‐SNHL, severe sensorineural hearing loss.

Survivors with s‐SNHL scored significantly lower than survivors without s‐SNHL on overall intellectual function (MD = −8.1, 95% CI = −10.0, −6.2; Figure S1), verbal reasoning (MD = −9.7 points, 95% CI = −11.4, −7.9; Figure S2), perceptual reasoning (MD = −6.6, 95% CI = −11.1, −2.1; Figure S3), working memory (MD = −5.3, 95% CI = −7.1, −3.4; Figure S4), processing speed (MD = −6.3, 95% CI = −9.92, −2.58; Figure S5), reading (MD = −6.2, 95% CI = −7.9, −4.5; Figure S6), and short‐term visual memory (MD = −6.0, 95% CI = −8.2, −3.7; Figure S7).

### 3.1 Sensitivity Analyses

The *Q* statistic of heterogeneity was below our threshold of *p* < 0.2 for perceptual reasoning (*I* ^*2*^ = 38.5%, *τ* ^2^ = 12.5, *p* = 0.15) and processing speed (*I* ^*2*^ = 45.3%, *τ* ^2^ = 8.29, *p* = 0.09). Subsequent sensitivity analyses revealed variability across the studies for perceptual reasoning (Table S4). The interpretation of the results did not change substantially with the systematic removal of studies.

The study by Bass et al. [3] was identified as a potential outlier for processing speed. While the mean difference in performance between survivors with and without s‐SNHL was somewhat attenuated when the Bass study was excluded, s‐SNHL was still significantly associated with reduced processing speed performance (MD = −4.2, 95% CI = −8.0, −0.5; Table S5).

## 4 Discussion

The main finding of the present systematic review and meta‐analysis, which included nine studies and more than 2000 survivors, was that childhood cancer survivors who experienced s‐SNHL demonstrated significantly lower performance across multiple neurocognitive domains. Our analyses suggest that survivors with s‐SNHL scored approximately 10 points lower on measures of verbal reasoning and six points lower on measures of reading, compared to those without. Discrepancies were not limited to domains that are relevant to language, however. Relative to those without s‐SNHL, survivors with s‐SNHL also performed approximately six points lower on measures of perceptual reasoning, working memory and processing speed. To contextualize these differences, mean performance of survivors without s‐SNHL was generally in the average to low average range relative to normative expectations, with some exceptions (Figure S1). Mean performance of survivors with s‐SNHL was lower—generally in the low average to below average range, approaching levels consistent with clinically meaningful impairment in some instances, and underscoring the pronounced cognitive vulnerability associated with hearing loss. By synthesizing data from multiple studies, our meta‐analysis provides a more comprehensive and precise evaluation of the true impact of ototoxicity on neurocognitive outcomes across heterogeneous patient populations, different cognitive measures, and various treatment protocols. Our results point to the urgent need for long‐term neuropsychological follow‐up in survivors of childhood cancer with s‐SNHL to ensure they receive appropriate support in school and/or the workplace.

### 4.1 Patient‐Reported Outcomes and SNHL

The cognitive burden associated with ototoxicity among childhood cancer is evident not only through standardized testing performance but also in self‐reported measures. For instance, the risk of impairment in task efficiency and memory was approximately 25% elevated among survivors of medulloblastoma with SNHL relative to those without SNHL [11]. Patient‐reported outcomes among adult survivors of pediatric sarcomas indicated that hearing loss was associated with higher rates of neurocognitive impairment [16, 25]. Likewise, parents of neuroblastoma survivors with hearing loss reported a higher incidence of learning disability and/or special educational needs than parents whose children did not develop hearing loss following neuroblastoma [14]. Although these results may contain potential recall bias, they provide valuable insights into survivors' experiences and quality of life.

### 4.2 Potential Mechanisms of SNHL Contributing to Cognitive Impairment

The mechanisms by which SNHL leads to cognitive impairment remain unclear, but several theories have been proposed [26]. One theory indicates that higher cognitive demand due to reduced auditory input drives more cognitive resources toward processing impaired sound, leaving fewer resources available for memory retention, attention, and executive function [27, 28]. A second theory is based on patients with Alzheimer's disease, where SNHL has been associated with temporal gray matter atrophy, which in turn affects cognitive functions [29]. A third theory posits that chronic auditory deprivation due to SNHL contributes to widespread neurodegeneration throughout the brain. These theories are primarily based on animal models and research in adult populations. Existing neuroimaging studies in children with SNHL have demonstrated alterations in regional brain volume and white matter microstructure relative to children without SNHL, suggesting that injury of auditory pathways may drive neural reorganization [30, 31, 32]. Given the known neurotoxicity of chemotherapy and/or radiation, the impact of treatment‐induced SNHL on the developing brain may be even more pronounced. Research is needed to more fully understand the mechanisms through which SNHL affects neurocognitive development among childhood cancer survivors and how factors such as age at diagnosis may contribute to the risk of neurocognitive difficulties related to SNHL.

### 4.3 Impact on Neurodevelopment Over Time

Hearing loss is not only a concern for immediate adverse effects in the context of cancer treatment, but can worsen over time, even after the treatment ends [33]. For instance, while 5% of patients reported ototoxicity at the conclusion of their chemotherapy regimen, approximately 44% of childhood cancer survivors developed clinically significant hearing loss more than 2 years posttreatment [33]. As hearing loss progresses over time, its effects are not limited to auditory deficits but also impact neurocognitive functions. Brennan‐Jones et al. [34] highlighted that survivors that sustained treatment‐related premature hearing loss within their first 3 years of life had disrupted speech and language acquisition, contributing to deficits in verbal reasoning, reading, and working memory—domains also significantly affected in our meta‐analysis. Moreover, hearing loss increases the burden on children in educational settings, due to challenges with academic performance and social–emotional functioning [35].

### 4.4 Limitations

While systematic pooling of the extant data on SNHL and neurocognitive performance in childhood cancer survivors is a major strength of the present study, various limitations should be noted. First, there was a considerable range in quality across the included studies, as assessed by a modified version of the Newcastle‐Ottawa Scale. We identified potential selection bias in some studies due to their reliance on retrospective, clinic‐referred samples. Other issues included low enrollment rates and/or did not offer comparisons between participants and nonparticipants. Some studies included a limited number of survivors with s‐SNHL or failed to comprehensively describe the comparability of hearing loss groups, such as by demographic or treatment characteristics. These limitations may partly stem from s‐SNHL not being a primary variable of interest in some of the studies [10, 12, 19, 36]. Further, all included studies were cross‐sectional in design, and the impact of SNHL on neurocognitive functioning over time among childhood cancer survivors remains an area in need of further exploration. Given that all available studies are observational, and most are cross‐sectional, the evidence should be interpreted with caution and cannot be considered conclusive [37].

Second, while most studies adjusted statistical models for relevant factors such as age at diagnosis and/or treatment characteristics, we could not extract these variables reliably across all studies. Due to the nature of standardized, neuropsychological testing, the extracted scores were all age‐adjusted; however, we cannot rule out that confounding contributed to the neurocognitive differences we observed between groups. Adding to this complexity, the heterogeneity of study designs, cancer types, length of follow‐up, and measurement procedures for determining hearing loss and/or neurocognitive performance may affect the validity of the findings.

Third, we initially sought to categorize data by treatment exposure (e.g., cranial radiation vs. chemotherapy) and age at diagnosis (i.e., survivors diagnosed < 5 years old vs. survivors diagnosed at ≥ 5 years old) to assess whether the impact of hearing loss on neurocognitive outcomes was different between these subgroups. Unfortunately, there was not sufficient data to conduct meaningful analyses. We attempted to compare neurocognitive outcomes of s‐SNHL and no/mild SNHL groups by demographic and treatment‐related factors (e.g., age at diagnosis, cranial RT), but inter‐study variability limited the interpretability of the results. A future analysis using individual‐level data may be more suited to investigating moderators of the effect of hearing loss on neurocognitive outcomes. Notably, the study by Bass and colleagues was the only one to stratify neurocognitive outcomes by exposure type (i.e., platinum‐only, cochlear RT, or neither), and they concluded that s‐SNHL is associated with neurocognitive deficits independent of the specific neurotoxic treatment received [3].

Finally, the generalizability of our findings may be limited by unmeasured factors that could influence neurocognitive outcomes (e.g., co‐occurring health conditions, socioeconomic status) ([18, 38, 39]) and by the inclusion of studies predating contemporary treatment approaches that incorporate otoprotective agents [40]. Despite these limitations, the present meta‐analysis has relevant clinical and practical implications in finding that sensorineural hearing loss poses a significant threat to cognitive functioning in childhood cancer survivors.

## 5 Conclusion

In summary, our findings support the importance of preventing ototoxicity secondary to cancer therapy, adhering to clinical practice guidelines for monitoring hearing loss in the context of treatment and survivorship to ensure rapid identification of hearing loss, and the need for neuropsychological assessment when s‐SNHL is identified. Reduced radiation exposures (e.g., proton radiotherapy) and further research into the efficacy of potential otoprotectants (e.g., sodium thiosulfate) may be avenues for preventing ototoxicity and SNHL in children [40, 41] undergoing treatment for CNS malignancies [10, 12, 19, 36]. Close monitoring of hearing loss in childhood cancer survivors should be prioritized, particularly given that some studies have found only 72% of those at risk have hearing tests during follow‐up, and fewer have full audiological monitoring [42]. While current guidelines recommend referral to speech‐language pathology and/or an educational liaison for survivors with SNHL, our findings suggest that neuropsychological assessment should be encouraged given the broad impact of s‐SNHL on domains beyond speech‐language to best identify cognitive/learning difficulties and support intervention development. These results specifically inform ongoing guideline development for ototoxicity surveillance and survivorship care by advocating for the routine inclusion of comprehensive neurocognitive screening protocols in survivors identified with significant SNHL. In terms of scientific implications, the consistent and significant impact observed across multiple neurocognitive domains urges further investigation into the underlying mechanisms driving these impairments. Understanding the relationship between age at exposure, treatment regimen, and ototoxicity and how these factors contribute to neurocognitive deficits could help develop better support for survivors with hearing loss and optimize neurocognitive outcomes.

## Author Contributions

**Jennifer E. Schlak:** data curation (equal), methodology (supporting), writing – original draft (lead), writing – review and editing (lead). **Tripti Shukla:** data curation (equal), methodology (supporting), writing – original draft (lead), writing – review and editing (lead). **Anne Eaton:** formal analysis (lead), visualization (lead), writing – review and editing (supporting). **Lindsay Blake:** data curation (supporting), methodology (supporting), software (supporting), writing – review and editing (supporting). **Alicia Kunin‐Batson:** conceptualization (lead), methodology (lead), project administration (lead), supervision (lead), writing – original draft (lead), writing – review and editing (lead). **Ellen van der Plas:** conceptualization (lead), methodology (lead), resources (lead), supervision (lead), writing – original draft (lead), writing – review and editing (lead).

## Ethics Statement

The authors have nothing to report.

## Conflicts of Interest

The authors declare no conflicts of interest.

## Supporting information


**Table S1:** Search strategy.
**Table S2:** Definition of severe sensorineural hearing loss across articles.
**Table S3:** Adapted version of the Newcastle‐Ottawa Scale to assess quality for cross‐sectional studies.
**Table S4:** Sensitivity analyses for perceptual reasoning.
**Table S5:** Sensitivity analyses for processing speed.
**Figure S1:** Full‐scale IQ. The top panel shows the forest plot and the bottom panel the funnel plot for full‐scale IQ.
**Figure S2:** Verbal reasoning. The top panel shows the forest plot and the bottom panel the funnel plot for verbal reasoning.
**Figure S3:** Perceptual reasoning. The top panel shows the forest plot and the bottom panel the funnel plot for perceptual reasoning.
**Figure S4:** Working memory. The top panel shows the forest plot and the bottom panel the funnel plot for working memory.
**Figure S5:** Processing speed. The top panel shows the forest plot and the bottom panel the funnel plot for processing speed.
**Figure S6:** Reading. The top panel shows the forest plot and the bottom panel the funnel plot for reading.
**Figure S7:** Short‐term visual memory. The top panel shows the forest plot and the bottom panel the funnel plot for short‐term visual memory.

## Data Availability

The data that supports the findings of this study are available in the Supporting Information of this article.
