# Supplementary material for: Association Between Sensorineural Hearing Loss and Neurocognitive Performance in Survivors of Childhood Cancer: A Systematic Review and Meta‐Analysis
Source: Cancer Med. 2025 Nov 20;14(22):e71394. doi: 10.1002/cam4.71394 (PMC12631741; doi:10.1002/cam4.71394)

Supplementary Materials

Association between sensorineural hearing loss and neurocognitive performance in survivors of childhood cancer: a systematic review and meta-analysis

# Supplementary Tables

## Supplementary Table 1 Search strategy

| **PubMed – June 29, 2023 – 287 results – updated to July 23, 2025 – 81 additional results** | | |
| --- | --- | --- |
| **Search #** | **Search Terms** | **# of Results** |
| **#1** | ("Ototoxicity"[Mesh] OR ototoxicity OR "Hearing Loss"[Mesh] OR “hearing loss” OR “hearing impairment” OR “sensorineural hearing loss” OR “snhl” OR "Hearing Loss, Sensorineural"[Mesh]) | **109,866** |
| **#2** | ("Antineoplastic Agents"[Mesh] OR "Antineoplastic Agents" [Pharmacological Action] OR "Radiotherapy"[Mesh] OR "Consolidation Chemotherapy"[Mesh] OR "Induction Chemotherapy"[Mesh] OR "Maintenance Chemotherapy"[Mesh] OR "Chemotherapy, Adjuvant"[Mesh] OR "Antineoplastic Protocols"[Mesh] OR chemotherapy OR radiotherapy OR radiation OR "Cisplatin"[Mesh] OR "Carboplatin"[Mesh] OR cisplatin OR carboplatin OR platinum-based chemotherapy) | **5,515,306** |
| **#3** | ("Cancer Survivors"[Mesh] OR cancer survivor* OR childhood cancer* OR pediatric cancer*) | **161,954** |
| **#4** | ("Neurocognitive Disorders"[Mesh] OR "Neuropsychological Tests"[Mesh] OR neuropsychological OR neurocognitiv*[tiab] OR "Neurobehavioral Manifestations"[Mesh] OR neurobehavioral OR "Neurodevelopmental Disorders"[Mesh] OR neurodevelopmental OR "Cognition/radiation effects"[Mesh] OR "Cognition Disorders"[Mesh] OR cognition OR "Educational Status"[Mesh] OR "Intelligence"[Mesh] OR "Intelligence Tests"[Mesh] OR intelligen*[tiab] OR intellectual* OR psychosocial[tiab] OR "Radiation Injuries"[Mesh] OR "Psychomotor Performance"[Mesh] OR "Psychomotor Disorders"[Mesh] OR psychomotor OR "Motor Skills"[Mesh] OR "Motor Skills Disorders"[Mesh] OR “motor skills” OR dexterity OR "Executive Function"[Mesh] OR “executive function” OR “executive ability” OR "Communication"[Mesh] OR communicat* OR "Comprehension"[Mesh] OR comprehend* OR "Thinking"[Mesh] OR think* OR dexterity OR "Psychophysiology"[Mesh] OR psychophysiology OR "Attention"[Mesh] OR attention OR "Reaction Time"[Mesh] OR “reaction time” OR "Memory Disorders"[Mesh] OR "Memory"[Mesh] OR memory OR "Perceptual Disorders"[Mesh] OR perceptual OR "Perception"[Mesh] OR perception OR "Problem Solving"[Mesh] OR “problem solving” OR "Learning"[Mesh] OR "Learning Disabilities"[Mesh] OR learning OR "Developmental Disabilities"[Mesh] OR “developmental disabilities” OR "Dyslexia"[Mesh] OR dyslexia OR "Language Disorders"[Mesh] OR "Language"[Mesh] OR language OR "Dyscalculia"[Mesh] OR dyscalculia OR "Judgment"[Mesh] OR judgement OR "Spatial Processing"[Mesh] OR “ spatial processing” OR “intellegence quotient” OR “IQ” OR "Emotional Regulation"[Mesh] OR "emotional regulation" OR "Impulsive Behavior"[Mesh] OR “impulsive behavior” OR "Spatial Behavior"[Mesh] OR “spatial behavior” OR "Reading"[Mesh] OR reading OR "cognitive processing speed" OR mathematic OR academic OR school) | **11,071,471** |
| **#5** | **#1 AND #2 AND #3 AND #4** | **292** |
| **#6** | English language limit | **287** |

## Supplementary Table 2 Definition of severe sensorineural hearing loss across articles

| Studies | Definition of severe SNHL | Parameters |
| --- | --- | --- |
| - Bass - Heitzer - Moxon-Embre - Orgel - Fay-McClymont - Conklin | Chang grade ≥2b | - >20 and <40 dB at 1000, 2000, or 3000 Hz - ≥40 dB at 2000 or 3000 Hz and above - ≥40 dB at 1000 Hz and above |
| - L’Hotta | SIOP Boston Ototoxicity Scale grade 3 or 4 | - >20 dB HL SNHL at 2000 Hz or 3000 Hz and above - >40 dB HL SNHL at 2000 Hz and above |
| - Notteghem | Major hearing deficit | - loss of ≥40 dB, between 2000 and 1000 Hz |
| - Tonning-Olson | Common Terminology Criteria for Adverse Events grades 2-4 | - Moderate to profound hearing loss - >40 dB |

## Supplementary Table 3 Adapted version of the Newcastle-Ottawa Scale to Assess Quality for Cross-Sectional Studies

| Criterion | Sub-criterion | Scoring |
| --- | --- | --- |
| Selection  (max 3 points) | Representativeness of the sample | - One point was given if the sample was truly representative of the average in the target population (all subjects or random sampling) or somewhat representative (non-random sampling) |
|  | Sample size | - One point was given if sample size was justified and satisfactory   - For single site studies, one point was given if ≥70% of eligible subjects were enrolled |
|  | Non-included subjects | - One point was given if comparability between included and non-included subjects was established, and if the inclusion rate was satisfactory. |
| Comparability  (max 2 points) | Subjects in different outcome groups are comparable, based on study design or analysis. | - One point was given if they compare the two groups on at least one demographic or treatment variable. |
|  | Confounding factors are controlled. | - One point was given if there was comprehensive comparison between both groups on at least one demographic and one treatment variables. |
| Outcome  (max 2 points) | Statistical tests | - One point was given if the statistical test used to analyze the data was clearly described and appropriate; - One point was given if measurement of the association was presented, including measures of variability (e.g., confidence intervals or SDs) and the probability level (p value). |

## Supplementary Table 4 Sensitivity analyses for perceptual reasoning

| **Removed** | **N Studies** | **N SNHL** | **N no SNHL** | **MD** | **95%CI** | **p** | **I^2^** |
| --- | --- | --- | --- | --- | --- | --- | --- |
| Tonning | 5 | 100 | 153 | -7.85 | -13.03, -2.67 | 0.0030 | 34.1 |
| Moxon-Emre | 5 | 80 | 251 | -6.49 | -11.99, -1.00 | 0.0206 | 50.4 |
| Notteghem | 5 | 113 | 213 | -7.84 | -12.62, -3.06 | 0.0013 | 32.4 |
| Orgel | 5 | 93 | 259 | -5.19 | -9.76, -0.61 | 0.0264 | 24.8 |
| Heitzer | 5 | 104 | 262 | -5.37 | -9.92, -0.83 | 0.0205 | 26.5 |
| Fay-McClymont | 5 | 115 | 267 | -6.97 | -11.92, -2.02 | 0.0058 | 49.3 |

SNHL: sensorineural hearing loss; MD=Mean difference; CI=Confidence Interval

## Supplementary Table 5 Sensitivity analyses for processing speed

| **Removed** | **N Studies** | **N SNHL** | **N no SNHL** | **MD** | **95%CI** | **p** | **I^2^** |
| --- | --- | --- | --- | --- | --- | --- | --- |
| Bass | 6 | 121 | 262 | -4.22 | -7.99, -0.46 | 0.02802 | 0.0 |
| Tonning | 6 | 446 | 1,265 | -6.40 | -10.55, -2.25 | 0.00250 | 48.8 |
| L'Hotta | 6 | 461 | 1,347 | -7.03 | -10.45, -3.60 | 5.9e-05 | 26.0 |
| Moxon-Emre | 6 | 421 | 1,355 | -7.00 | -10.94, -3.07 | 0.00048 | 39.2 |
| Orgel | 6 | 442 | 1,372 | -5.87 | -10.06, -1.67 | 0.00612 | 54.0 |
| Heitzer | 6 | 450 | 1,374 | -5.71 | -10.13, -1.29 | 0.01140 | 53.7 |
| Fay-McClymont | 6 | 461 | 1,383 | -6.59 | -10.24, -2.95 | 0.00039 | 48.9 |

SNHL: sensorineural hearing loss; MD=Mean difference; CI=Confidence Interval

# Supplementary Figures

## Supplementary Figure 1 Full-scale IQ. The top panel shows the forest plot and the bottom panel the funnel plot for full-scale IQ.


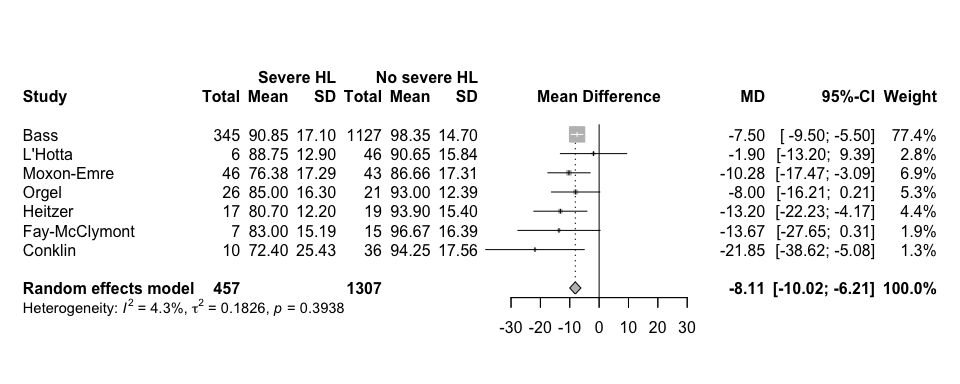


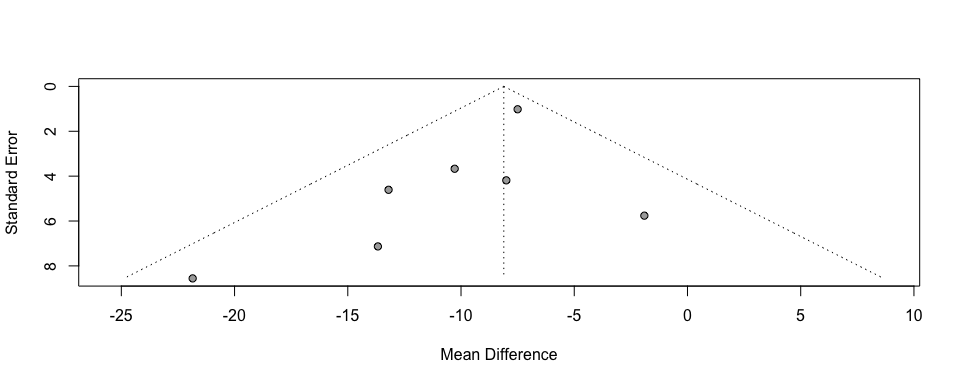


## Supplementary Figure 2 Verbal reasoning. The top panel shows the forest plot and the bottom panel the funnel plot for verbal reasoning.


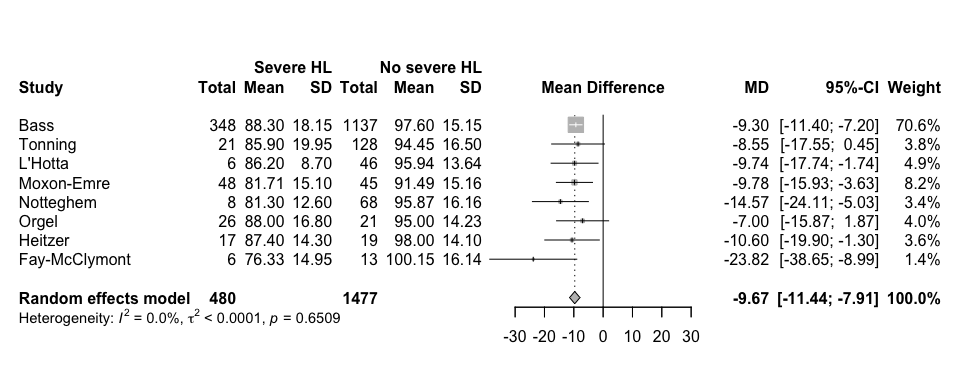

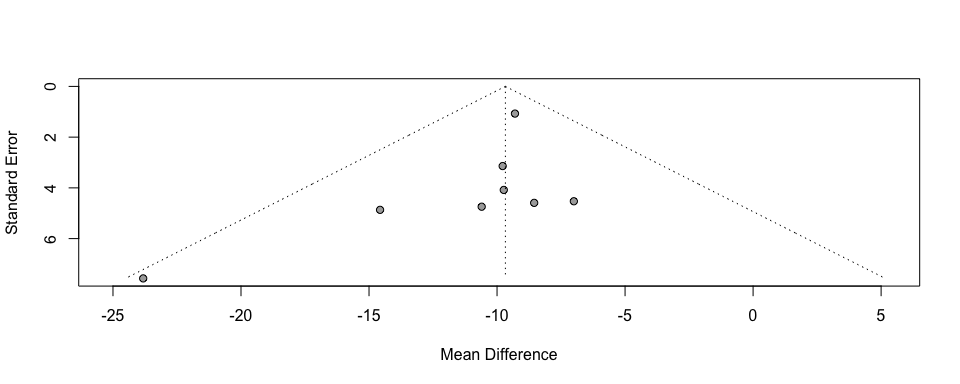


## Supplementary Figure 3 Perceptual reasoning. The top panel shows the forest plot and the bottom panel the funnel plot for perceptual reasoning.


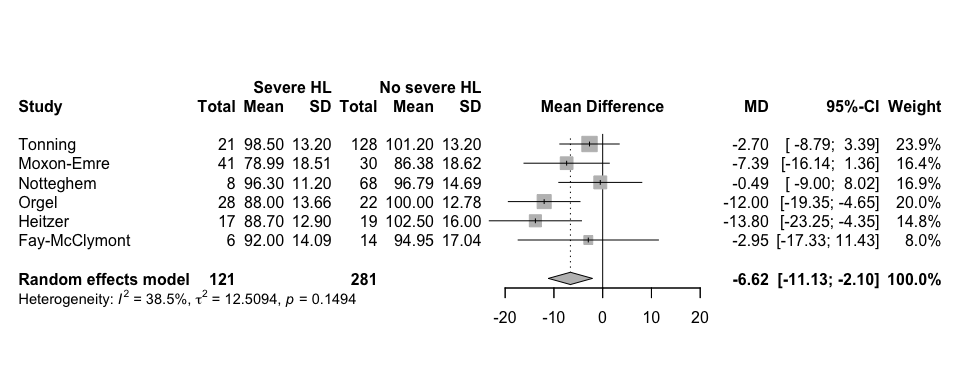

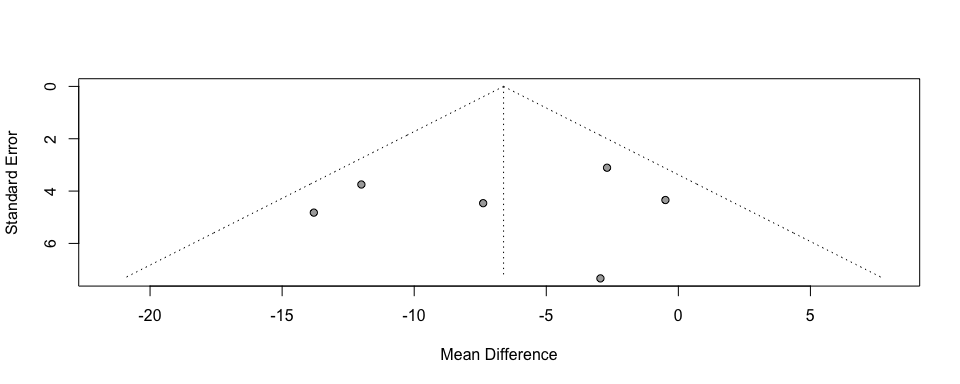


## Supplementary Figure 4 Working memory. The top panel shows the forest plot and the bottom panel the funnel plot for working memory.


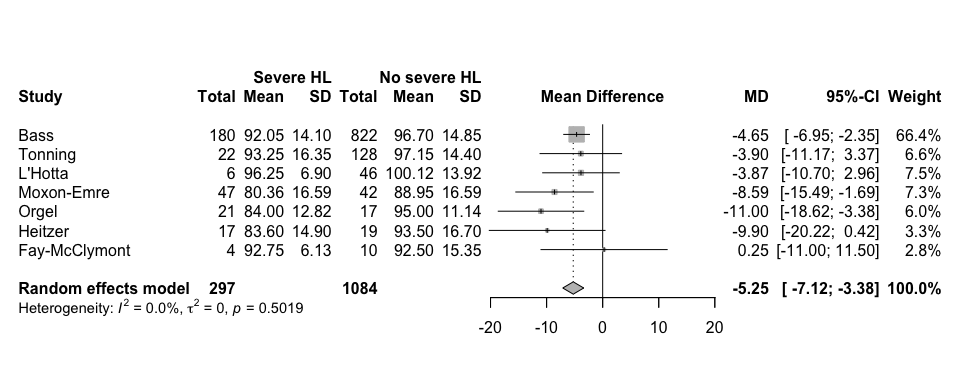

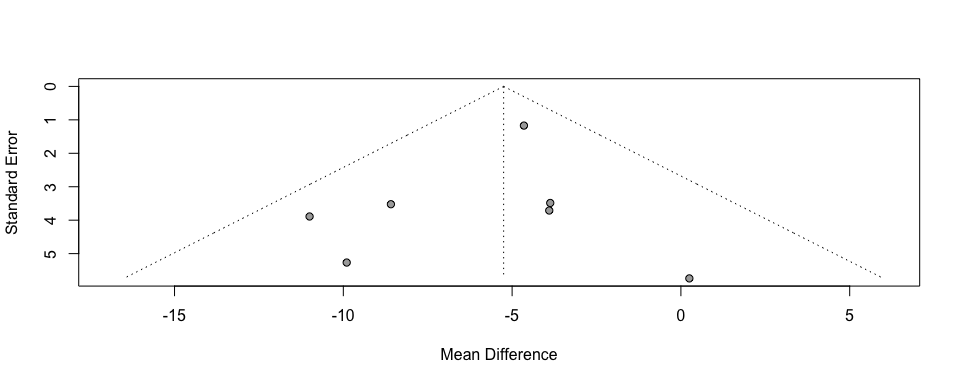


## Supplementary Figure 5 Processing speed. The top panel shows the forest plot and the bottom panel the funnel plot for processing speed.


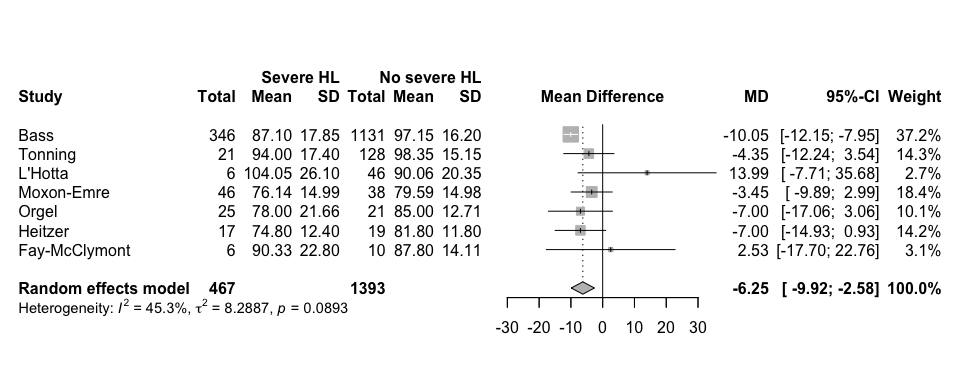

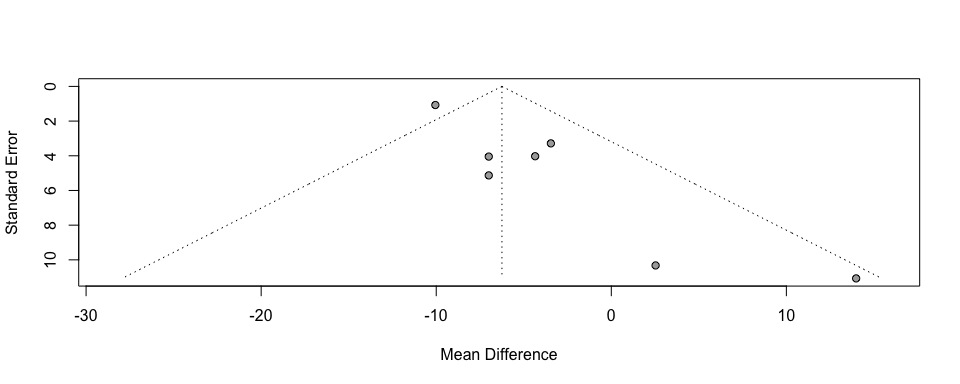


## Supplementary Figure 6 Reading. The top panel shows the forest plot and the bottom panel the funnel plot for reading.


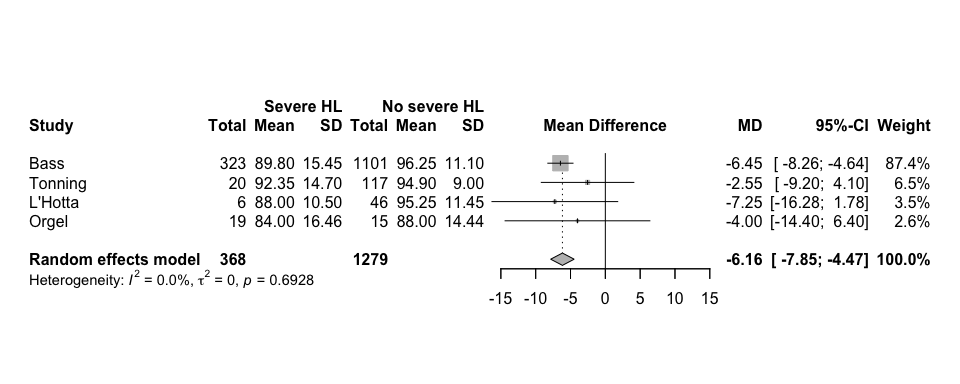

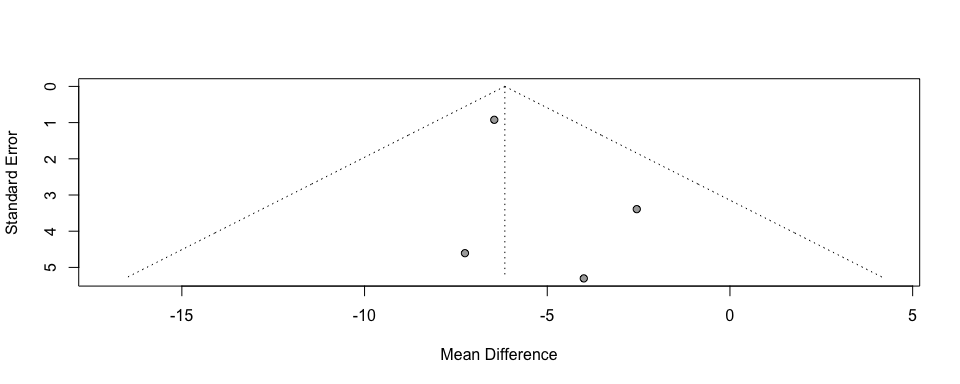


## Supplementary Figure 7 Short-term visual memory. The top panel shows the forest plot and the bottom panel the funnel plot for short-term visual memory.


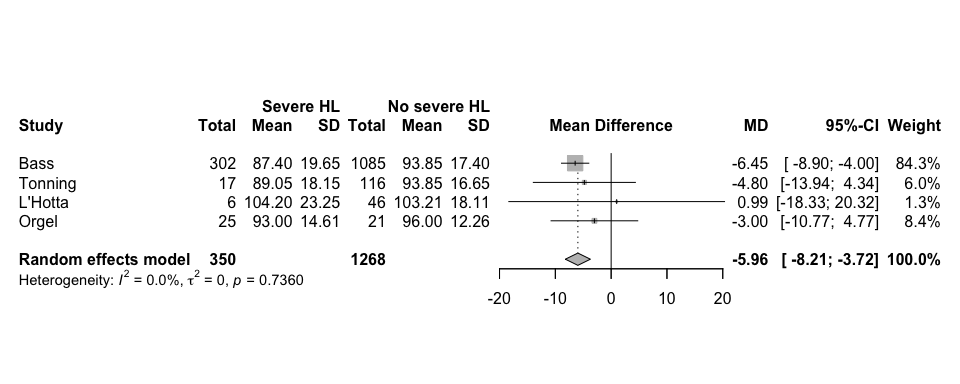

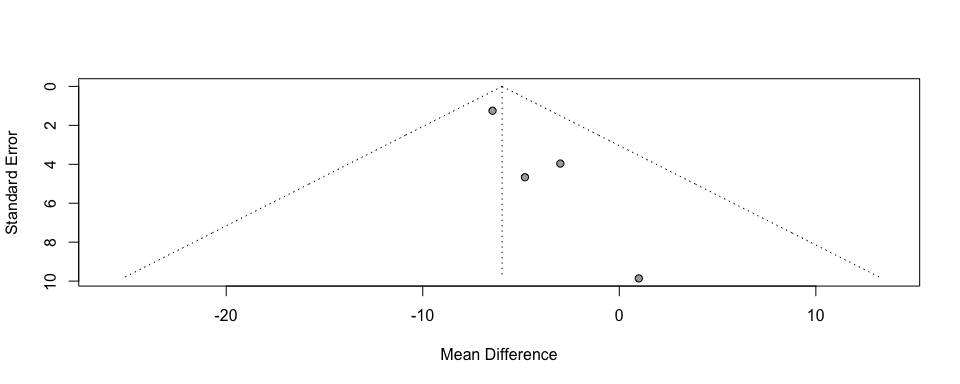

Supplement: Supplementary file 1 — Table S1: Search strategy. Table S2: Definition of severe sensorineural hearing loss across articles. Table S3: Adapted version of the Newcastle‐Ottawa Scale to assess quality for cross‐sectional studies. Table S4: Sensitivity analyses for perceptual reasoning. Table S5: Sensitivity analyses for processing speed. Figure S1: Full‐scale IQ. The top panel shows the forest plot and the bottom panel the funnel plot for full‐scale IQ. Figure S2: Verbal reasoning. The top panel shows the forest plot and the bottom panel the funnel plot for verbal reasoning. Figure S3: Perceptual reasoning. The top panel shows the forest plot and the bottom panel the funnel plot for perceptual reasoning. Figure S4: Working memory. The top panel shows the forest plot and the bottom panel the funnel plot for working memory. Figure S5: Processing speed. The top panel shows the forest plot and the bottom panel the funnel plot for processing speed. Figure S6: Reading. The top panel shows the forest plot and the bottom panel the funnel plot for reading. Figure S7: Short‐term visual memory. The top panel shows the forest plot and the bottom panel the funnel plot for short‐term visual memory. [file CAM4-14-e71394-s001.docx]
